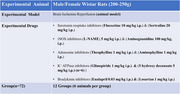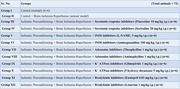# Evaluation of the Possible Mechanisms of Action of Brain Ischemic Pre‐conditioning in Rats

**DOI:** 10.1002/alz70857_096767

**Published:** 2025-12-24

**Authors:** Priyanka Chandolia

**Affiliations:** ^1^ Centre of Excellence in Pharmaceutical Sciences, Guru Gobind Singh Indraprastha University, Dwarka (New Delhi) India, NAJAFGARH, New Delhi, India

## Abstract

**Background:**

Ischemic preconditioning (IPC) refers to the phenomenon where brief, non‐lethal episodes of ischemia protect the brain against subsequent ischemic injury. This study aims to explore the potential mechanisms underlying IPC‐induced neuroprotection in rats.

**Method:**

Male and Female Wistar rats were subjected to global cerebral ischemia using the four‐vessel occlusion method. IPC was induced by subjecting animals to 5 minutes of ischemia followed by 24 hours of reperfusion before a subsequent longer ischemic insult. Behavioral, biochemical, and histological analyses were performed to evaluate the neuroprotective effects of IPC. Key parameters assessed included infarct volume, oxidative stress markers (malondialdehyde and superoxide dismutase), pro‐inflammatory cytokines (TNF‐α and IL‐6), and the expression of endogenous neuroprotective molecules such as brain‐derived neurotrophic factor (BDNF) and heat shock protein 70 (HSP70).

**Result:**

Rats preconditioned with IPC showed significantly reduced infarct volume and improved neurobehavioral outcomes compared to controls. IPC significantly attenuated oxidative stress, evidenced by decreased malondialdehyde levels and increased superoxide dismutase activity. Moreover, IPC downregulated pro‐inflammatory cytokines and upregulated BDNF and HSP70 expression in the ischemic brain. These findings suggest that IPC confers neuroprotection via modulation of oxidative stress, inflammatory pathways, and induction of endogenous protective proteins.

**Conclusion:**

Brain ischemic preconditioning in rats triggers multifaceted protective mechanisms, including antioxidant, anti‐inflammatory, and neurotrophic pathways. Understanding these mechanisms could provide valuable insights for developing novel therapeutic strategies to mitigate ischemic brain injury in humans.

**Keywords**: ischemic preconditioning, neuroprotection, oxidative stress, inflammation, brain ischemia, rats